# Nanofabricated Ultraflexible Electrode Arrays for High‐Density Intracortical Recording

**DOI:** 10.1002/advs.201700625

**Published:** 2018-03-10

**Authors:** Xiaoling Wei, Lan Luan, Zhengtuo Zhao, Xue Li, Hanlin Zhu, Ojas Potnis, Chong Xie

**Affiliations:** ^1^ Department of Biomedical Engineering The University of Texas at Austin Austin TX 78712 USA; ^2^ Department of Physics The University of Texas at Austin Austin TX 78712 USA

**Keywords:** electron‐beam lithography, flexible neural electrodes, high‐density intracortical recording, in vivo extracellular recording, nanofabrication

## Abstract

Understanding brain functions at the circuit level requires time‐resolved simultaneous measurement of a large number of densely distributed neurons, which remains a great challenge for current neural technologies. In particular, penetrating neural electrodes allow for recording from individual neurons at high temporal resolution, but often have larger dimensions than the biological matrix, which induces significant damage to brain tissues and therefore precludes the high implant density that is necessary for mapping large neuronal populations with full coverage. Here, it is demonstrated that nanofabricated ultraflexible electrode arrays with cross‐sectional areas as small as sub‐10 µm^2^ can overcome this physical limitation. In a mouse model, it is shown that these electrodes record action potentials with high signal‐to‐noise ratio; their dense arrays allow spatial oversampling; and their multiprobe implantation allows for interprobe spacing at 60 µm without eliciting chronic neuronal degeneration. These results present the possibility of minimizing tissue displacement by implanted ultraflexible electrodes for scalable, high‐density electrophysiological recording that is capable of complete neuronal circuitry mapping over chronic time scales.

## 1 Introduction

Implanted neural probes^1^, ^2^ such as microwires,^3^ tetrodes,^4^ and silicon‐based microelectrodes^5^, ^6^, ^7^ are among the most important techniques in both fundamental and clinical neuroscience. Scientifically, they remain our only option to temporally resolve the fastest electrophysiological activities of individual neurons, which provides critical information to dissect the neural circuitry.^8^, ^9^, ^10^, ^11^ Clinically, neural electrodes have been successfully used in the treatment for a number of neurological disorders^12^, ^13^ such as Parkinson's disease,^14^ epilepsy,^15^ and obsessive compulsive disorder.^12^ Moreover, they allow for direct communication between brain and man‐made devices, which can enable applications such as human brain–machine interface and neuroprosthetics.^16^, ^17^ However, these conventional neural electrical probes typically have dimensions substantially larger than neurons and capillaries, which fundamentally precludes the possibility of interrogating the whole neuronal population in a functional brain region. In particular, microwire electrodes,^3^ tetrodes,^4^ and Utah array^18^ host only one recording site at the tip of each wire, and therefore cannot simultaneously record neural activity at multiple depths. Micro‐electromechanical system‐based silicon probes^19^, ^20^ have significantly increased the number of recording sites on one probe. However, these silicon probes typically have cross‐sectional areas around or greater than 10^3^ µm^2^, which gives the volume per electrode similar to that of the microwire and tetrodes, all at two orders of magnitude larger than the average size of a neuronal soma (Table S1, Supporting Information). In addition, because these probes are strongly invasive to living brain tissue,^21^, ^22^ to maintain tissue vitality their highest implantation density is limited by only allowing at most 1–2% of the enclosed volume to be occupied by the electrode array.^23^ Therefore, the smallest interprobe spacing is limited to be at least several hundred micrometers for both microwire array and silicon probes.^24^ Although flexible neural electrodes are generally believed to induce less tissue reaction than their rigid counterparts, their highest implantation densities demonstrated to date are still at similar values.^25^, ^26^

Previous efforts have demonstrated that smaller electrodes composed of both novel^27^, ^28^ and conventional materials^29^ resulted in suppressed inflammatory responses that can potentially support higher density implantation than larger electrodes. In the effort of increasing packing density on one neural probe, advanced lithography techniques such as electron‐beam lithography (EBL) have been used to fabricate silicon‐based microelectrodes that enable closely packed recording sites along the length of the shank.^30^, ^31^, ^32^, ^33^ However, despite great capacity and potential, these nanofabricated probes based on conventional rigid architectures are known to elicit significant reactions by the host tissue, leading to challenges in high‐density implantation over chronic time scale.^31^ Recently developed ultraflexible nanoelectronic neural probes,^34^, ^35^, ^36^, ^37^ using a substrate‐less, multilayer layout, markedly reduced the cross‐sectional area to the subcellular range,^36^ but demonstrated limited electrode density along the probe due to the fabrication resolution of the planar photolithography techniques. In this paper, we combined the unconventional ultraflexible device architecture with state‐of‐the‐art EBL to fabricate the nanoelectronic thread (NET‐e) probe with densely packed electrode arrays and versatile design patterns. We fabricated neural probes each hosting eight or more electrodes with substantially reduced cross‐sectional areas at below 10 µm^2^. Taking advantage of sub‐10 µm shuttle devices, we further demonstrated implantation of NET‐e probes with interprobe spacing as small as 60 µm, which overcame the dimensional limits of electrodes for high‐density neural mapping.

## 2 Results and Discussion

**Figure 1:**
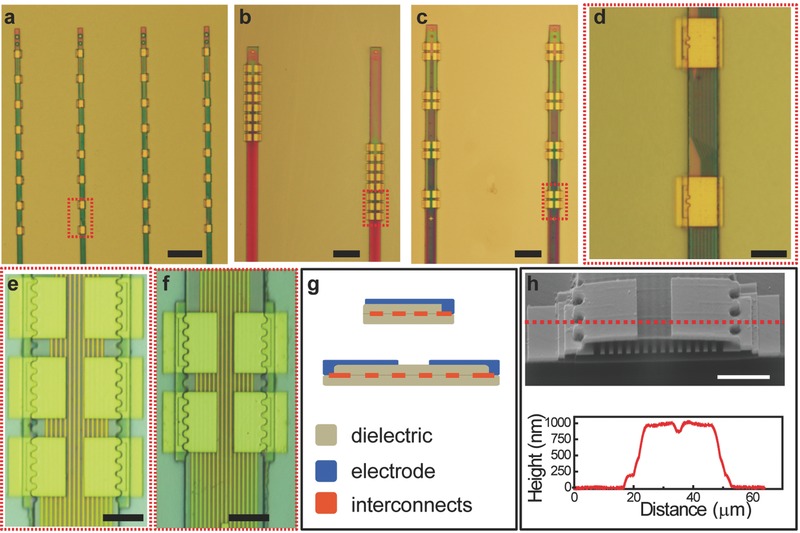
As‐fabricated NET‐e devices. a–c) Photographs of the EBL section of a variety of NET‐e devices, including a) linear array NET‐e‐l, b) oversampling array NET‐e‐o, and c) tetrode‐like array NET‐e‐t. d–f) Zoom‐in images of panels (a)–(c) as marked by the dashed boxes, showing the fine structure and precise interlayer alignment. g) Sketch of the two multilayer architecture of the NET‐e devices. h) Scanning electron microscopy (SEM) image (top) and height profile by atomic force microscopy (AFM, bottom) of an NET‐e‐t electrode showing the sub‐micrometer thickness and fine layered structures at the cross section. Dashed line marks the position of the AFM height measurement. Scale bars: 50 µm, panels (a)–(c); 10 µm, panels (d–f); and 5 µm, panel (h).

We adopted a hybrid method involving both electron beam and optical lithography^38^ to fabricate NET‐e devices with high throughput (detailed fabrication procedures in Section S1 and Figure S1 in the Supporting Information). We used EBL to define the implanted section (the “thread”) where dimension constraints were stringent and used photolithography with relaxed resolution requirement for larger structures that were not implanted into brain tissue (an example of EBL and photolithography sections are shown in Figure S2 in the Supporting Information). **Figure** **1**a–f shows the overview and zoom‐in images of the as‐fabricated EBL section on NET‐e probes with different patterns of electrode arrays. NET‐e‐l (Figure 1a) hosted a linear array of electrodes with a cross‐sectional profile of 0.8 µm × 8 µm, the smallest among all reported electrodes to our knowledge (Figure S3 and Table S1, Supporting Information). NET‐e‐t (Figure 1b) was designed to function similarly as multiple tetrodes spanning across the cortical depth, hosting groups of four closely spaced electrodes every 100 µm along the thread. NET‐e‐o (Figure 1c) had a continuum of individually addressed electrodes along the thread. For improved single‐unit detection and sorting yield, both NET‐e‐t and NET‐e‐o were designed to have electrode spacing smaller than their detection range to enable spatial oversampling in action potential recording. To minimize NET‐e probe's cross‐sectional area, we designed a multilayer architecture with no substrate similar to previous work,^36^ where interconnect traces and electrodes were fabricated on different layers separated by insulating layers (Figure 1g,h). To facilitate the transition from EBL to optical lithography and to avoid disconnected interconnects and insulating layers, we intentionally overlapped the sections of EBL and photolithography by at least 4 µm in all directions in all layers as shown in Figure S2 (Supporting Information).

We successfully overcame several technical challenges in the fabrication of NET‐e probes, including precise interlayer alignment across the entire wafer, and the application of SU‐8 as a sensitive negative e‐beam resist to construct the insulation layers (discussed in details in the following sections). We achieved a minimum linewidth of 200 nm (pitch of 400 nm) for the interconnect traces and sub‐100 nm interlayer alignment. The total thickness of the NET‐e probes was determined mostly by the thickness of the two SU‐8 layers, and was precisely controlled to be 0.8–1 µm (Figure 1h) by fine tuning the e‐beam exposure dose of SU‐8 with 0.5 µC cm^−2^ accuracy (Figure S4, Supporting Information).

**Figure 2:**
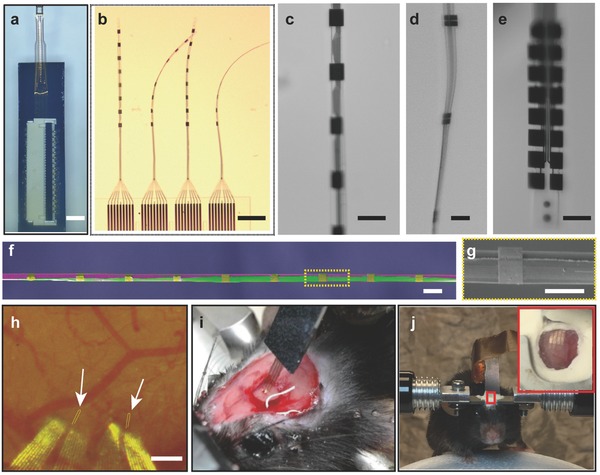
Implantation of the NET‐e devices. a–e) Photograph of NET‐e devices immersed in water: a) overview of an NET‐e device including the carrier chip and a connector to external I/O mounted atop; b) the e‐beam section of four threads on panel (a) showing the ultraflexibility; c–e) electrodes on c) NET‐e‐l, d) NET‐e‐t, and e) NET‐e‐o. f) Pseudocolor SEM image of an NET‐e‐l device (in purple and gold) attached on a shuttle device made of carbon fiber (purple) with a diameter of 7 µm, showing the small dimension of both. g) Zoom‐in view in panel (f). h) Photograph showing an NET‐e‐o probe successfully delivered into living mouse brain. Arrows denote the delivery entry sites. Dashed lines mark the probe implanted beneath the brain surface. Image was taken 20 d post implantation through a cranial optical window. i) Photograph of a mouse immediately after NET‐e probe implantation. Gently pulling the carrier chip straightened the relaxed section of the probe without pulling out the implanted section. j) Photograph of a head‐constrained mouse on a custom‐built treadmill for awake recording. Scale bars: 2 mm, panel (a); 200 µm, panels (b,h); 20 µm, panels (c–f); and 10 µm, panel (g).

The sub‐1 µm thickness led to ultraflexibility that precludes free standing in air after being released from the substrates. **Figure** **2**a–e shows the entire device (Figure 2a), the flexible section (Figure 2b), and the EBL sections of the NET‐e probes (Figure 2c–e) floating in water after released. Because the ultraflexible NET‐e devices could not self‐support for insertion into brain tissue, we used a microshuttle device and a “needle‐and‐thread” strategy to deliver the NET‐e probes to the desired location and depth in the mouse brain. Similar to the delivery of larger NETs we recently reported,^36^ the temporary engagement during delivery was enabled by a micropost machined at the end of the shuttle device fitting into a microhole fabricated at the end of the NET‐e probe (Figure S5, Supporting Information). Significantly different from their larger counterparts,^36^ the much reduced dimension of the NET‐e imposed a more stringent requirement on the shuttle device dimension. The ultrasmall NET‐e probes were only successfully delivered using shuttle devices with diameters smaller than 10 µm (Figure 2f,g), whereas for shuttle devices with larger diameters, the capillary force when retracting the shuttle device was sufficiently large to drag out the NET‐e probe. We attribute it to the much‐larger‐than‐probe acute tissue displacement during implantation, which led to insufficient friction force by the surrounding tissue to retain the probe in place during shuttle device retraction, a failure mode that was previously reported for larger shuttle devices and flexible neural probes.^39^ The use of microshuttle devices and NET‐e smaller than 10 µm brought down the surgical footprints to subcellular dimensions, which formed tight tissue integration immediately after implantation in which the NET‐e remained embedded in brain tissue even when gently pulled (Figure 2h,i). We implanted NET‐e devices into somatosensory cortex in the mouse brain at targeted depths of 500–700 µm at the tip so that the electrodes along the probe span over the cortical depth. The implantation yield was 60% (*n* = 15). In vivo recording was typically performed at 1–2 months after implantation. Taking advantage of the ultraflexibility and the ultrasmall dimensions, we accommodated chronic optical access concurrently with NET‐e implants (Figure 2j) similarly as we recently demonstrated.^36^

**Figure 3:**
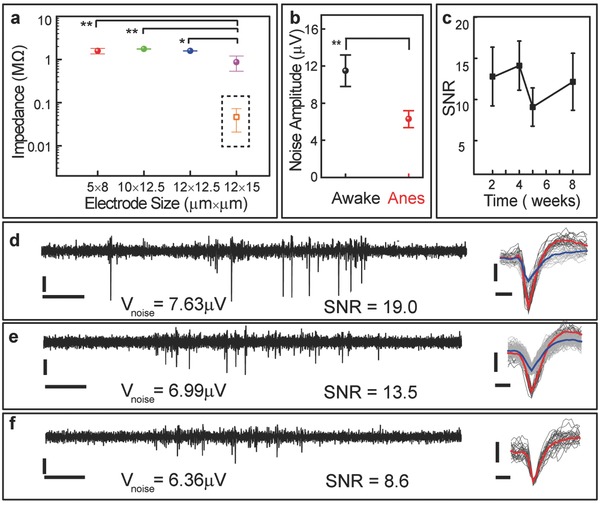
In vivo recording performance of NET‐e devices. a) In vivo impedance at 1 kHz measured at 1week after implantation, *n* = 20 for each dimension. Dashed box: impedance of electrodes with PEDOT coating (*n* = 6) measured in 1 × PBS. b) In vivo noise level of the smallest electrode (5 µm × 8 µm) at anesthetized (right, 6.46 µV median) and awake (left, 11.4 µV median) measurements (bandwidth: 0.5 Hz to 7.5 kHz), *n* = 20. Measurement time: 5 weeks after implantation. c–f) SNR of action potential recordings by NET‐e probes (*n* = 10 electrode sites of three NET‐e‐l probes in three anesthetized mice) over c) 8 weeks and representative recordings from three implanted NET‐e‐l electrodes in an anesthetized mouse d) 2 weeks and e,f) 5 weeks after implantation. Left: 1s real‐time recording trace; 250 Hz high‐pass filter applied. Right: Superimposed spikes isolated from the recording traces. All unit events were plotted in light gray and averaged waveforms plotted in red and blue. SNR was calculated using the larger waveform when there were two recorded on one electrode. Vertical scale bars: 50 µV, panels (c–e); horizontal scale bars: 0.1 s, panels (c–e, left) and 0.25 ms (c–e, right). The symbols * and ** in panels (a,b) denote significant difference of *p* < 0.01 and *p* < 0.001 between the groups, respectively.

The electrodes on NET‐e probes were finished by gold thin film and had dimensions ranging from 5 × 8 to 12 × 15 µm^2^ depending on the design. The impedance at 1 kHz ranged between 1 and 1.8 MΩ, larger than their photolithography‐defined counterparts,^36^ which is consistent with previous results^40^ (**Figure** **3**a). While reducing the dimensions of the neural electrodes is beneficial for recording density and specificity, as well as tissue compatibility, it leads to the increase of impedance and therefore the decrease of signal‐to‐noise ratio (SNR). Conducting polymers such as poly(3,4‐ethylenedioxythiophene) (PEDOT),^41^, ^42^, ^43^, ^44^, ^45^, ^46^, ^47^ poly(pyrrole),^48^ nanomaterials such as carbon nanotubes,^49^ silicon nanowires,^50^ and polymer nanotubes,^51^ and other functional materials such as IrO*_x_* 1, 52 have been used to decrease electrode impedance and increase charge injection capacity. We electrochemically deposited PEDOT on the NET‐e electrodes with an area of 12 × 15 µm^2^ and observed the impedance decreased from 0.87 ± 0.33 to 0.046 ± 0.026 MΩ (Figure 3a, dashed box). The decrease of impedance is expected to improve the SNR and suppress low‐frequency artifact.^51^ In the rest of this work, we only used electrodes without coating for consistency, whose relatively large impedance combined with animal motion during awake measurements led to slightly elevated noise levels, 11.4 ± 1.7 µV (Figure 3b), but all these electrodes were capable of detecting and isolating single‐unit action potentials. Figure 3c shows the SNR of *n* = 10 electrode sites that yielded detectable spikes in three probes implanted in three mice for 8 weeks. Figure 3d–f shows the representative recordings, the SNR, and the spike waveforms that were isolated from the 10 min recording session from three different electrodes at 2 weeks (Figure 3d) and 5 weeks (Figure 3e,f). The recording in Figure 3d had well‐isolated single units and was among the best in signal quality with SNR of 19.0 and a noise level of 7.63 µV. The recording in Figure 3f had discriminable spike clusters, but was among the worst in signal quality with an SNR of 8.6 and an average noise level of 6.36 µV. The SNR is slightly lower but on par with that from its microfabricated counterparts^36^ and other conventional bare electrodes without polymer coating,^51^, ^53^ which is consistent with their reduced electrode dimension.

**Figure 4:**
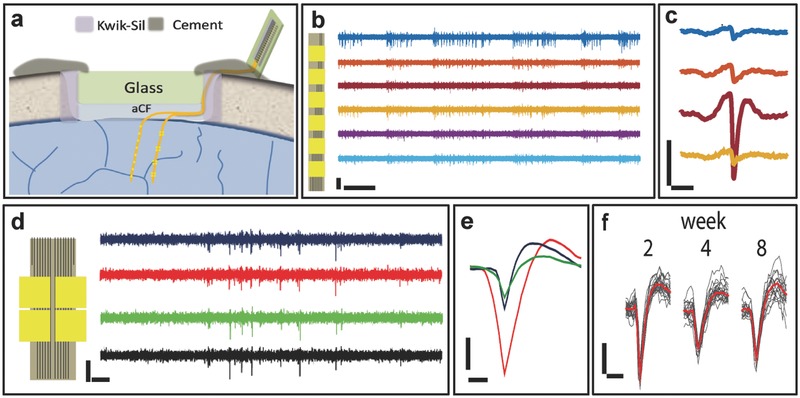
Representative unit recording from NET‐e electrodes. a) Schematic showing the relative position of electrodes on NET‐e‐l (left) and NET‐e‐t (right) devices in the brain. b) Recording time trace from an NET‐e‐l probe hosting a linear electrode array. Inset sketches the NET‐e‐l probe. c) 10 ms recording trace from panel (b) on the top four electrodes highlighting a single spike that was picked up by adjacent electrodes with attenuated amplitudes. Same color code as panel (b). b,c) The recording was performed 5 weeks after implantation. d) Recording time trace from a group of four NET‐e‐t electrodes showing correlated spikes. Inset sketches the NET‐e‐t probe. e) Sorted single‐unit waveforms from panel (d). d,e)The recording was performed 9 weeks after implantation. f) Superimposed spikes isolated from the same NET‐e‐l electrode over 8 weeks post implantation. Averaged waveforms shown in red. Vertical scale bars: 50 µV, panels (b–f); horizontal scale bars: 0.1 s, panels (b) and (d); 2 ms, panel (c); and 0.5 ms, panels (e,f).

**Figure** **4** shows the representative recording from NET‐e‐l electrodes and NET‐e‐t electrodes, performed 5 and 9 weeks after implantation, respectively. We used the adjacent electrodes in the two designs to estimate their spike detection range, which depends on its impedance, dimension, and geometry. The detection range is a crucial parameter to determine the necessary electrode density for achieving complete detection of all enclosed neurons within a brain region.^8^, ^23^ As shown in Figure 4b,c, on NET‐e‐l electrodes that were separated by 80 µm along the thread, the same spikes were typically picked up by adjacent recording sites with strongly attenuated amplitudes, suggesting that the detection range is comparable to the electrode separation. Consistent with this detection range, the NET‐e‐t electrodes recorded temporally correlated spikes from the four “tetrode‐like” electrodes in one group which were densely packed within an area of 18 µm × 12 µm (Figure 4d,e). The spatial–temporal correlation of the four electrodes improved the detection and sorting fidelity of single‐unit action potentials (Figure 4e) analogous to tetrodes.^4^ In addition, this scalable architecture enabled simultaneous detection at multiple brain depths.

**Figure 5:**
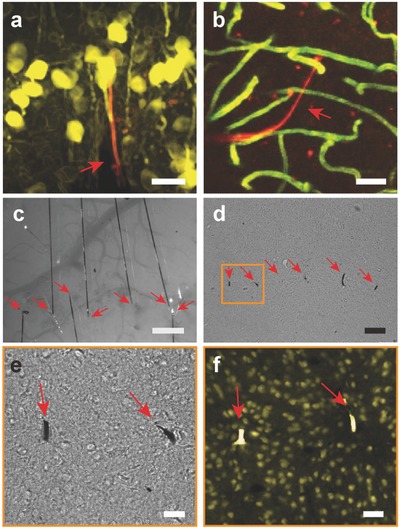
In vivo and postmortem tissue–probe interface. a) Reconstruction of in vivo 2P images of neurons (yellow, Thy1‐YFP) surrounding an NET‐e‐l probe (red) 2 months post implantation. Image stack: 100–320 µm below the brain surface. b) 3D reconstruction of vasculature by in vivo 2P microscopy around NET‐e‐l probe (red) 2 months postimplantation, showing normal capillary networks (green). Image stack: 100–320 µm below the brain surface. c) Photograph showing in vivo implantation of multiple NET‐e‐l probes. Arrows denote the implantation locations. d) Bright field image of a postmortem tissue slice at the probe–tissue interface as shown in panel (c) 4 months postimplantation. Arrows denote the probes. e) Zoom‐in bright field image of the boxed region shown in panel (d). Arrows denote the probes. f) Fluorescence image of the same area as in panel (e). Color code: yellow, NeuN, labelling neuron nuclei; Rhodamine 6G, labelling NET‐e probe. Normal neuronal density was observed near the two probes at inter‐probe distance was 60 µm. Scale bars: 50 µm, panels (a–d); 10 µm, panels (e,f).

Our previous publication^36^ demonstrated that microfabricated NETs afford stable recording and nondegrading tissue–probe interface in mice through a collection of comprehensive characterizations. In this work, we did not include systematic studies on the chronic performance of NET‐e probes because they share similar materials, structures, and the ultraflexibility. We therefore expect NET‐e probes have comparable tissue–probe interface and recording stability as previously demonstrated NETs. In the recording period of this study (2 months), we did not observe chronic deterioration in recording performance (Figure 4f). Similar to the observation of larger NETs,^36^ in vivo two‐photon (2P) imaging 2 months after implantation showed normal neuronal density (**Figure** **5**a) and morphology of vasculature (Figure 5b) near the NET‐e electrodes. Importantly and advantageous to the previous larger NET devices, the much reduced lateral dimensions of NET‐e probes allowed for multiprobe implantation at a previously unattainable high density. Figure 5c shows the photograph of living brain immediately after implantation of seven NET‐e probes, where the interprobe distance was as small as 60 µm, a distance significantly smaller than the pitch of current state‐of‐the‐art microelectrode arrays^31^ and would allow electrodes on the two adjacent probes to have overlapping detection range. We examined postmortem tissue–probe interface at 4 months after implantation (Figure 5d–f). The bright‐field images (Figure 5d,e) and the fluorescent images from immunohistological staining (Figure 5f) revealed healthy tissue morphology and normal neuronal density, suggesting that the long‐term presence of NET‐e device arrays at high packing density did not result in “dead” zone near the electrodes. This is also consistent with our previous study where an unintentionally folded NET probe at a curvature of about 50 µm in living brain did not elicit astrocytes accumulation at 3.5 months postimplantation.^36^

Different components of the NET‐e probes require different EBL techniques to construct, which were individually optimized for high yield, throughput, and fabrication resolution. Standard EBL using poly(methyl methacrylate) (PMMA) as the positive resist followed by metallization and lift‐off was performed to define the interconnects and the electrodes. The highest resolution was required for interconnect traces, which is determined by the EBL sample stage stitching error, because the length of the thread (about 1 mm) was much larger than the size of the writing field in the EBL tool used in this work (80 µm). Nonstandard EBL was performed to construct the insulation layers, for which we tested both PMMA^54^ and SU‐8^55^ as negative EBL resists. These two resists were chosen for their good insulation, excellent tensile strength after hard baking, and tunable thickness depending on the exposure doses.^54^, ^55^ Because PMMA requires a high dose of about 40 000 µC cm^−2^ to crosslink^54^ that leads to prohibitively long EBL exposure times even at high currents, we chose to use SU‐8 for higher fabrication throughput.

Our fabrication yield was about 70% with little variation among different design patterns that required fabrication resolution ranging between 200 and 400 nm. We have thoroughly examined our fabrication process and identified that the yield loss is mainly due to fabrication defects. All fabrication procedures, including four EBL steps, seven photolithography steps, and five metal deposition steps, were performed manually, which make it difficult to completely avoid microparticles and scratches especially on the long, narrow interconnect traces. The EBL exposure time was relatively short. For example, an entire 4″ wafer that contains 8 NET‐e devices and a total of 256 electrodes typically requires exposure times of ≈5 min for the insulating layers composed of SU‐8, 2 h for the interconnects, and 15 min for the electrode layers. However, the fabrication throughput was mostly limited by the two‐step iterative alignment process during EBL that was necessary for sub‐100 nm registration accuracy across the entire wafer. Despite the relatively low throughput compared with photolithography, the application of EBL greatly improved the fabrication resolution and reduced the probe's overall dimensions, and further offers great flexibility in patterning nanoscale structures by design. We are confident that the fabrication yield, throughput, and resolution can be further improved using more advanced EBL equipment.

Ultraflexibility requires additional efforts in careful handling and implantation. However, ultraflexibility is necessary to achieve stable chronical recording and nondegrading tissue–probe interface.^36^ Significantly different from our previous work that focused on the chronically stable recording and nondegrading tissue–probe interface, this work focused on pushing the dimensional limit of ultraflexible neural electrodes on a similar device architecture. Using nanofabrication and refined implantation techniques, we further reduced both the device cross‐sectional area and the surgical footprints per recording site by an order of magnitude. Importantly and advantageous to the previous larger NET devices, the much reduced dimensions of NET‐e probes allowed for multiprobe implantation at a previously unattainable high density (e.g., 60 µm interprobe distance) without eliciting observable neuronal death over chronic implantation. The capability of fabricating scalable, subcellular‐sized electrodes with an ultraflexible architecture represents, in our opinion, a crucial first step toward long‐term, full‐coverage mapping of the neural activity in a sizable brain region.

## 3 Conclusion

In this study, we demonstrated the possibility of integrating neural electrode arrays within a subcellular form factor and their implantation in a high‐density, scalable manner. By applying nanofabrication techniques on unconventional substrate‐less design of neural probes, we drastically reduced the physical dimensions of neural probes. Combining these nanofabricated ultraflexible probes with minimally invasive implantation methods at subcellular surgical footprints, we developed a practical approach to overcome current physical limits in the design and implantation of intracortical neural electrodes, which paves the road for chronic, full‐coverage, neural recording and complete circuit‐level mapping of neural activity.

## 4 Experimental Section

*Shuttle Device Fabrication and Assembly*: A straight segment of carbon fiber was attached to a stainless steel microneedle (prod# 13561‐10, Ted Pella, Inc.) for convenient handling. It was then cut to the designed length (2–3 mm) using focused ion beam (FIB). An anchor post was micromilled at the tip of the shuttle device using FIB to shape a well‐defined micropost (≈3 µm in diameter, 4 µm in height).

*Electrochemical Deposition of PEDOT*: PEDOT deposition was carried out in 3,4‐ethylenedioxythiophene monomer (EDOT) (0.07 m) and KNO_3_ (0.13 m) aqueous solution. The electrochemical deposition was performed by a WaveNano USB Potentiostat (Pine Research Instrumentation) via its interface software, the AfterMath, at room temperature. PEDOT was deposited in potentiostatic mode at 0.8 V, with a two‐electrode configuration. The working electrodes were connected to the electrode site. The reference electrode (Ag/AgCl electrode) and counter electrode were connected to a platinum wire in contact with the EDOT/KNO_3_. The polymerization time was typically set to 20 s and can be varied to control the coating thickness.

*Animals and Surgery*: Wild‐type male mice (C57BJ/6, 8 weeks old, Taconic) were used in the experiments. Mice were housed at the Animal Research Center, UT Austin (12 h light/dark cycle, 22 °C, food and water ad libitum).

Mice were anesthetized using isoflurane (3% for induction and maintained at 1–2%) in medical‐grade oxygen. The skull was exposed and prepared by scalping the crown and removing the fascia, and then was scored with the tip of a scalpel blade. A 3 mm × 3 mm square craniotomy was performed with a surgical drill over the somatosensory cortex. Dura mater was carefully removed to facilitate the delivery. After NET‐e probe implantation (described in the following section), the remaining flexible segment of the NET probe, which connected the bonding pad on the substrate with the electrodes inside the brain, was routed to the edge of the cranial opening. The exposed brain was then protected by artificial cerebrospinal fluid (ACSF) and a coverslip #1 (Fisher Scientific) fit into the cranial opening. The space between the coverslip and the remaining skull was filled with Kwik‐sil adhesive (World Precision Instruments). After the skull was cleaned and dried, a layer of low‐viscosity cyanoacrylate was applied over the skull. An initial layer of C&B‐Metabond (Parkell Inc.) was applied over the cyanoacrylate and the Kwik‐sil. A second layer of Metabond was used to cement the coverslip and the NET carrier chip to the skull. All procedures complied with the National Institute of Health guidelines for the care and use of laboratory animals and were approved by the University of Texas Institutional Animal Care and Use Committee.

*Neural Probe Implantation—Insertion*: In typical procedures, a flexible section of NET‐e probe was placed on the brain surface where dura mater was removed. The shuttle device was mounted on a micromanipulator (MP‐285, Shutter instrument) vertically, and positioned atop the engaging hole at the end of the probe. As the shuttle device traveled downward, the anchor post entered into the hole and pulled the neural probe into the brain tissue. Once the neural probe reached the desired depth, the shuttle device was retracted, and the neural probe was released and left embedded in the brain tissue.

*Two‐Photon Imaging*: 2P imaging was performed in a laser scanning microscope (Praire Technology) equipped with a 20× water immersion objective (NA 1.0; Zeiss) and a Ti:sapphire excitation laser (MaiTai DS, Spectra‐Physics) at a few weeks to months postimplantation. The laser was tuned to 810–910 nm for 2P excitation (power: 3.0–50 mW, dwell time: 4.0–6.0 µs). Fluorescence emissions were detected simultaneously by two standard photomultiplier tubes with a 595/50 nm filter (Semrock, US) for “red” fluorescence emission and a 525/70 nm filter (Semrock, US) for “green” fluorescence emission. Mice were anesthetized using isoflurane (3% for induction and 1.5% during experiment) in medical‐grade oxygen to maintain full immobility during imaging and placed in a frame that stabilized the head on the microscope stage. Anesthetized animals were given fluorescein isothiocyanate (FITC)‐dextran (0.1 mL, 5% w/v, Sigma) retro‐orbitally to label blood vessels prior to imaging. To facilitate imaging the probe–tissue interface beyond superficial cortical layers, the NET‐e probes were doped with sulforhodamine 6G (Sigma) in the insulating layers and delivered at about 45° with respect to the skull.

*Awake Electrophysiological Recording*: Mice were allowed a 1–2 week recovery period after surgery. For recording, mice were head constrained on a custom‐made air‐supported spherical treadmill to allow walking and running, similar to the setup previously used for optical imaging.^44^ The treadmill was made of an 8″ diameter Styrofoam ball (Floracraft) levitated by a thin cushion of air between the ball and a casting containing air jets. Voltage signals from the NEC devices were amplified and digitized using a 32‐channel RHD 2132 evaluation system (Intan Technologies) with a bare Ag wire inserted into the contralateral hemisphere of the brain as the grounding reference. The sampling rate was 20 kHz, and a 300 Hz high‐pass and a 60 Hz notch filter were applied for single‐unit recording. Mice and the amplifier were placed in a noise‐attenuated, electrically shielded chamber. Impedance of the recording electrodes was measured using the same setup at 1 kHz prior to recording.

*Histological Sample Preparation*: Mice were given lethal intraperitoneal injections of 0.15 mL ketamine mixed with xylazine (10 mg mL^−1^ xylazine in 90 mg mL^−1^ ketamine) and then perfused intracardially with oxygenated, cold (≈4 °C) modified ACSF (2.5 × 10^−3^ m KCl, 1.25 × 10^−3^ m NaH_2_PO_4_, 25 × 10^−3^ m NaHCO_3_, 0.5 × 10^−3^ m CaCl_2_, 7 × 10^−3^ m MgCl_2_, 7 × 10^−3^ m dextrose, 205.5 × 10^−3^ m sucrose, 1.3 × 10^−3^ m ascorbic acid, and 3.7 × 10^−3^ m pyruvate) followed by 4% paraformaldehyde in 0.02 m phosphate‐buffered saline (PBS). Brains were cryoprotected in a 30% sucrose/4% paraformaldehyde solution overnight. Tissue was sectioned into 20–50 µm slices perpendicular to the probe using a Leica CM1950 cryostat (Leica Microsystems). The slices were washed (3 × 5 min) and incubated in hot sodium citrate solution (850–950 °C, 0.01 m in H_2_O) for 30 min for antigen retrieval. Then, the slices were washed (3 × 5 min), incubated in blocking solution and permeabilized (0.5% Triton X and 10% normal goat serum (Sigma) in PBS) for 3 h at room temperature, washed (4 × 5 min), and incubated in fluorophore conjugated antibodies for 24 h at 4 °C. Reagents used for Neurons were (Millipore): Alexa Fluor 488 conjugated anti‐NeuN antibody, clone A60.

*Statistical Analysis*: Standard statistical analysis was performed using Matlab to calculate the average and standard deviation for the impedance, SNR, noise amplitude for the recording data. Analysis of variance (ANOVA) was done on the impedance with different electrode sizes after implantation. Independent *t*‐test was performed on the results of noise amplitude for awake and anesthetized in vovo recording (Matlab).

## Conflict of Interest

The authors declare no conflict of interest.

## Supporting information

Supplementary
