# Supplementary material for: Nanofabricated Ultraflexible Electrode Arrays for High‐Density Intracortical Recording
Source: Adv Sci (Weinh). 2018 Mar 10;5(6):1700625. doi: 10.1002/advs.201700625 (PMC6010728; doi:10.1002/advs.201700625)
Supplement: Supplementary file 1 — Supplementary [file ADVS-5-1700625-s001.pdf]

Copyright WILEY-VCH Verlag GmbH & Co. KGaA, 69469 Weinheim, Germany, 2018.

ADVANCED SCIENCE
Open Access

Supporting Information

for *Adv. Sci.,* DOI: 10.1002/advs.201700625

Nanofabricated Ultraflexible Electrode Arrays for High-Density Intracortical Recording

*Xiaoling Wei, Lan Luan, Zhengtuo Zhao, Xue Li, Hanlin Zhu, Ojas Potnis, and Chong Xie**

Copyright WILEY-VCH Verlag GmbH & Co. KGaA, 69469 Weinheim, Germany, 2017.

Supporting Information

Nanofabricated ultraflexible electrode arrays for high-density intracortical recording

Xiaoling Wei[†], *Lan Luan*[†], *Zhengtuo Zhao*[†], *Xue Li, Hanlin Zhu, Ojas Potnis, Chong Xie*[*]

Dr. X. Wei, Dr. L. Luan, Z. Zhao, X. Li, H. Zhu, O. Potnis, Prof. C. Xie

Department of Biomedical Engineering

The University of Texas at Austin

78712 Austin, TX, U.S.A.

E-mail: chongxie@utexas.edu

Dr. L. Luan

Department of Physics

The University of Texas at Austin

78712 Austin, TX, U.S.A.

Dr. X. Wei, Dr. L. Luan, Z. Zhao

These authors contribute equally to this work

1. Fabrication process flow:

The fabrication of NET-e devices was carried out on 100 mm silicon wafers (900 nm SiO_2, n-type 0.005 V·cm, University Wafer). Key fabrication steps are showed in Figure S1 and are outlined as follows: (i) EBL and metallization of 100 nm Au to define a set of global and chip alignment marks for precise alignment among different fabrication steps. (ii) Photolithography and electron beam deposition were used to pattern a 100-nm nickel metal layer as the sacrificial layer that will be etched to release the flexible sections of the device at the end of the fabrication process. (iii) A 300- to 500-nm layer of SU-8 photoresist (2000.5; MicroChem Corp.) was deposited over the entire wafer and patterned by photolithography and EBL to define an insulating layer. Figure S1 shows the design of the device and marks the sections for photolithography and EBL. (iv) Photolithography and metallization of 100 nm Au was used to define the contact pads and interconnects outside the thread. (v) EBL and metallization was used to define the interconnects within the thread. Symmetrical Cr/Au/Cr or Cr/Pd/Cr (2 – 3 / 60 – 100 / 2 – 3 nm) metal was sequentially deposited to control the stress. (vi) Another 300- to 500-nm layer of SU-8 photoresist was deposited over the entire chip and patterned by photolithography and EBL to define the top insulating layer. (vii) EBL and electron beam deposition of Cr/Au (2 – 3 /100 nm) were used to define and deposit the electrodes that are individually addressed by contact pads through interconnects. (viii) The wafer was hard-baked at 180 – 190 °C for 5 hours to improve adhesion between two SU-8 layers and then was cut to individual devices using dicing saw (ADT, 7100 series). (ix) After fabrication, a 33-pin flexible flat connectors (FFC) (series 502598, Molex) was mounted on the matching contact pads on the Si substrate. (x) The flexible section was released from the substrate by etching off the nickel layer (Nickel Etchant TFB; Transene Company Inc.) for 2-4 h at 25 °C, rinsed and stored in distilled (DI) water before use.

2. Calibration of EBL dose for SU-8 photoresist:

When using SU-8 as negative tone EBL resist[1], the dose is about a few μC/cm^2 (ca. 1% of PMMA dose) and is highly sensitive to proximity effect that changes with substrates, the resist thickness, the pattern design and the acceleration voltage of the EBL[1-3]. In addition, the thickness of exposed SU-8 (2000.5, Microchem) after development also depends on the exposure dose[3]. We therefore fine tune the dose to achieve targeted thickness post-exposure while maintaining ca. 100 nm fabrication resolution, in order to resolve the micron-size engaging hole for delivery and to realize sharp edge profile for the thread, so that sufficient insulation was achieved for interconnectors defined in proximity of the thread edge. As shown in Figure S4, the optimal dose is 2.0 μC/cm^2 (writing current: 50 pA; acceleration voltage: 50 kV) for NET-e width of 5 – 20 μm. The final thickness of the exposed SU-8 is above 300 nm for all width.

Supporting table:

Neural probe type	Flexible Probe?	Typical dimensions	Probe Cross-section (A) (μm^2)	Total surgical footprint (μm^2)	# of electrodes (n)	Cross-section per electrode (μm^2)	Reference
Silicon microelectrode	No	15 μm, 60 μm (t,w)	900	900	4	225	Kipke[4]
	No	15 μm, 55 μm (t,w)	825	825	4	206	Vetter[5]
	No	15 μm, 90 μm (t,w)	1350	1350	4	338	Ludwig[6]
	No	23 μm, 85 μm (t,w)	1960	1960	64	30.5	Du[7]
	No	23 μm, 60 μm (t,w)	1380	1380	32	43.1	Du[7]
	No	23 μm, 40 μm (t,w)	920	920	16	57.5	Du[7]
	No	15 μm, 83 μm (t,w)	1250	1250	8	156	Gillespie[8]
	No	15 μm, 50 μm (t,w)	750	750	128	5.86	Scholvin[9]
	No	15 μm, 50 μm (t,w)	750	750	200	3.75	Scholvin[9]
Microwire	No	50 μm (d)	1960	1960	1	1960	Nicolelis[10-11]
	No	30-50 μm (d)	707-1960	707-1960	1	707-1960	Schwarz[12]
Tetrode	No	4 x 12 μm (d)	452	452	4	452	Gray[13]
	No	4 x 12.5 μm (d)	491	491	4	491	Rothschild[14]
Carbon fiber electrode	No	8.6 μm (d)	58.1	58.1	1	58.1	Kozai[15]
Polyimide probe	Yes	(10-20) μm, 160 μm (t,w)	1600-3200	80002	2	800-1600	Rousche[16]
	Yes	10 μm, 536 μm (t,w)	5360	16000	8	670	Felix[17]
	Yes	12.5 μm, 196 μm (t,w)	2450	8000	16	153	Kozai[18]
	Yes	25 μm,10 mm (t,w)	250000	12150000	360	694	Viventi[19]
Parylene-C	Yes	20 μm, 35 μm (t,w)	700	40000	3	233	Sohal[20]
	Yes	15 μm, 300 μm (t,w)	4500	50000	8	563	Kim[21]
	Yes	15 μm, 10 μm (t,w)	1500	Not mentioned	10	150	Roger[22]
Polyester	Yes	20 μm, 400 μm (t,w)	8000	100000	1	8000	Kim[23]
SU8	Yes	1 μm, 1 mm (t,w)	1000	8000 – 30000	19	111	Xie[24]
	Yes	1 μm, 2 mm (t,w)	2000	7000 – 30000	16	125	Liu[25]
	Yes	1 μm, 50 μm (t,w)	50	90 – 140	8	6.25	Luan[26]
	Yes	1.5 μm, 10 μm (t,w)	15	50 – 70	2	7.50	Luan[26]

	Yes	0.8 µm, 8 µm (t,w)	6.4	40 – 50	8	0.80	This work
	Yes	1 µm, 15 µm (t,w)	15	50 – 70	16	0.94	This work
	Yes	1 µm, 28 µm (t,w)	28	60 – 80	16	1.75	This work

Table. S1: Comparison of representative neural probes with current study in dimensions, number of electrodes, cross-section per electrode and implantation footprint. For probes of a shaft shape, probe cross-section is $A = w \times t$, where w is the width and t is the thickness. For cylindrical probe, probe dimensions are $A = (\pi d^2)/4$, where d is the diameter of the probe. The cross-section per electrode is expressed as A/n, where n is the number of electrodes on each probe. The total surgical footprint includes the extra tissue displacement associated with implanting flexible probes in addition to the probe's cross-section.

Supporting figures:

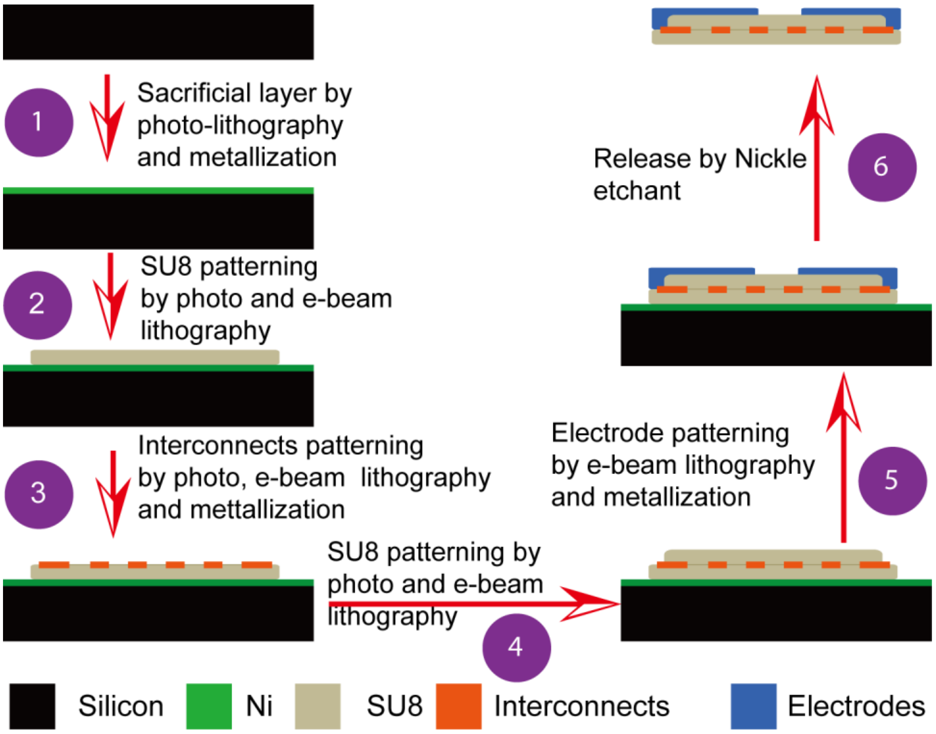

Figure S1: Flow chart of the NET-e probe fabrication process.

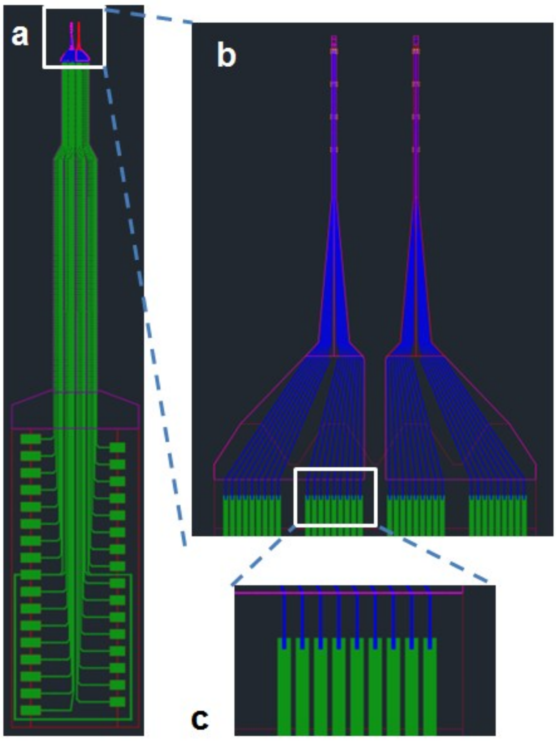

Figure S2: Design drawings of a typical NET-e device. (a) the overview of the device. Green section was patterned by photolithography while blue and purple section inside the box was patterned by EBL. (b) zoom-in view of the EBL section in the box in (a). Solid box marks the transition region between photolithography and EBL shown in (c). (c) zoom-in view of the transition region. The EBL patterned interconnects overlaps with photolithography defined interconnects in all four directions to reduce disconnection due to alignment errors.

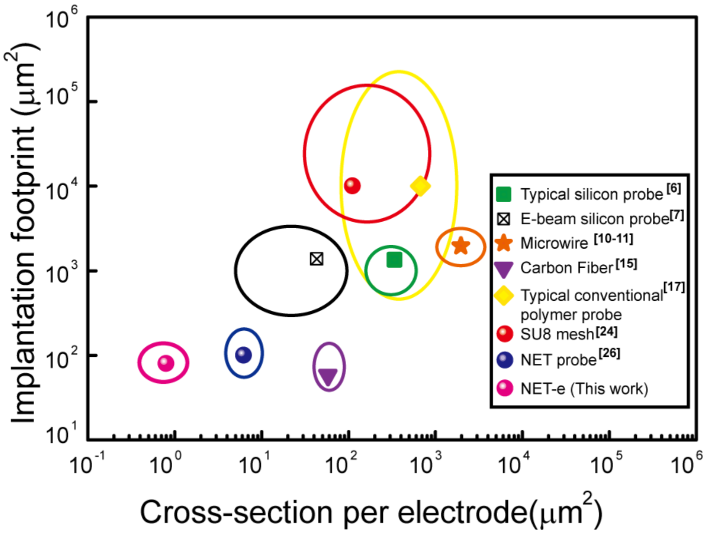

Figure S3: Drastically reduced implantation footprint and cross-section per electrode (*A* / *n*) of NET-e probes compared with previously reported neural probes. Symbols: representative probes for each category of probes. Ovals: range of values in implantation footprint and cross-section per electrode for the same category of probes.

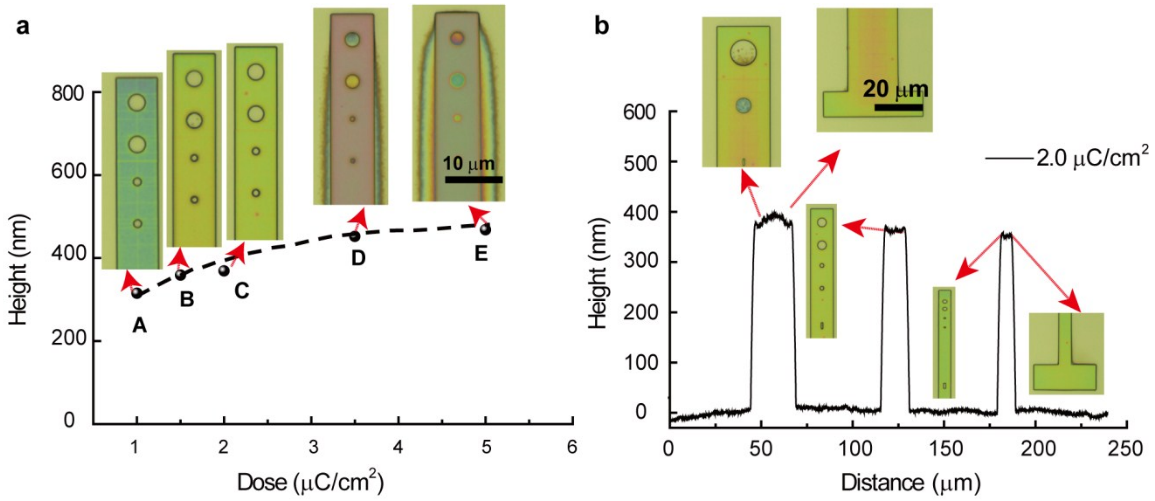

Figure S4: Dose calibration of EBL for SU-8 layers, using ribbons of a selection of width and microscale holes with varying diameters (smallest feature at 1 μm). (a) The dependence of the post-exposure height on EBL dose. Insets show the post-exposure patterns at the matching doses. The thickness increased with exposure dose up to 2.5 μC/cm^2, but fabrication resolution was compromised at higher dose as evidenced by strong halo around the ribbon and partially or fully blocked micro-holes on the ribbon. We therefore determined the optimal dose to be 2.0 μC/cm^2. (b) Heights of post-exposure SU-8 film for 5, 10 and 20 μm wide ribbons at 5 different doses, confirming that 2.0 μC/cm^2 can be used for different designs of the NET-e devices.

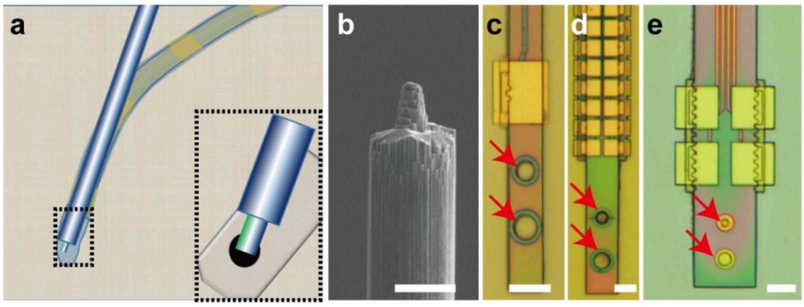

Figure S5: Implantation of NET-e devices. (a) schematics of the shuttle-device assisted implantation. Insets shows the temporary engagement scheme. (b) SEM image of a typical shuttle device made of carbon fiber, scale bar: 5 µm. (c – e) photographs of the as-fabricated NET-e devices highlighting the engaging micro-hole for implantation in NET-e-l (c), NET-e-o (d) and NET-e-t (e) devices. Scale bars: 10 µm.

References for supporting information:

[1] A. K. Nallani, S. W. Park, J. B. Lee, *Proc. SPIE* **2003**, *5116*, 414.

[2] A. Pepin, V. Studer, D. Decanini, Y. Chen, *Microelectron. Eng.* **2004**, *73-4*, 233.

[3] B. Bilenberg, S. Jacobsen, M. S. Schmidt, L. H. D. Skjolding, P. Shi, P. Boggild, J. O. Tegenfeldt, A. Kristensen, *Microelectron. Eng.* **2006**, *83*, 1609.

[4] D. R. Kipke, R. J. Vetter, J. C. Williams, J. F. Hetke, IEEE Trans. *Neural Syst. Rehabil. Eng.* **2003**, *11*, 151.

[5] R. J. Vetter, J. C. Williams, J. F. Hetke, E. A. Nunamaker, D. R. Kipke, *IEEE Trans. Biomed. Eng.* **2004**, *51*, 896.

[6] K. A. Ludwig, J. D. Uram, J. Y. Yang, D. C. Martin, D. R. Kipke, *J. Neural. Eng.* **2006**, *3*, 59.

[7] J. Du, T. J. Blanche, R. R. Harrison, H. A. Lester, S. C. Masmanidis, *PLoS One* **2011**, *6*, e26204.

[8] A. K. Gillespie, E. A. Jones, Y. H. Lin, M. P. Karlsson, K. Kay, S. Y. Yoon, L. M. Tong, P. Nova, J. S. Carr, L. M. Frank, Y. D. Huang, *Neuron* **2016**, *90*, 740.

[9] J. Scholvin, J. P. Kinney, J. G. Bernstein, C. Moore-Kochlacs, N. Kopell, C. G. Fonstad, E. S. Boyden, *IEEE Trans. Biomed. Eng.* **2016**, *63*, 120.

[10] M. A. Nicolelis, D. Dimitrov, J. M. Carmena, R. Crist, G. Lehew, J. D. Kralik, S. P. Wise, *Proc. Natl. Acad. Sci. U S A* **2003**, *100*, 1104.

[11] M. A. Nicolelis, A. A. Ghazanfar, B. M. Faggin, S. Votaw, L. M. Oliveira, *Neuron* **1997**, *18*, 529.

[12] D. A. Schwarz, M. A. Lebedev, T. L. Hanson, D. F. Dimitrov, G. Lehew, J. Meloy, S. Rajangam, V. Subramanian, P. J. Ifft, Z. Li, A. Ramakrishnan, A. Tate, K. Z. Zhuang, M. A. L. Nicolelis, *Nat. Methods* **2014**, *11*, 670.

[13] C. M. Gray, P. E. Maldonado, M. Wilson, B. McNaughton, *J. Neurosci. Methods* **1995**, *63*, 43.

[14 G. Rothschild, E. Eban, L. M. Frank, *Nat. Neurosci.* **2017**, *20*, 251.

[15] T. D. Y. Kozai, N. B. Langhals, P. R. Patel, X. P. Deng, H. N. Zhang, K. L. Smith, J. Lahann, N. A. Kotov, D. R. Kipke, *Nat. Mater.* **2012**, *11*, 1065.

[16] P. J. Rousche, D. S. Pellinen, D. P. Pivin, J. C. Williams, R. J. Vetter, D. R. Kipke, *IEEE Trans. Biomed. Eng.* **2001**, *48*, 361.

[17] S. H. Felix, K. G. Shah, V. M. Tolosa, H. J. Sheth, A. C. Tooker, T. L. Delima, S. P. Jadhav, L. M. Frank, S. S. Pannu, *J. Vis. Exp.* **2013**, *79*, e50609.

[18] T. D. Kozai, D. R. Kipke, *J. Neurosci. Methods* **2009**, *184*, 199.

[19] J. Viventi, D. H. Kim, L. Vigeland, E. S. Frechette, J. A. Blanco, Y. S. Kim, A. E. Avrin, V. R. Tiruvadi, S. W. Hwang, A. C. Vanleer, D. F. Wulsin, K. Davis, C. E. Gelber, L. Palmer, J. Van der Spiegel, J. Wu, J. L. Xiao, Y. G. Huang, D. Contreras, J. A. Rogers, B. Litt, *Nat. Neurosci.* **2011**, *14*, 1599.

[20] H. S. Sohal, A. Jackson, R. Jackson, G. J. Clowry, K. Vassilevski, A. O'Neill, S. N. Baker, *Front. Neuroeng.* **2014**, *7*, 10.

[21] B. J. Kim, J. T. Kuo, S. A. Hara, C. D. Lee, L. Yu, C. A. Gutierrez, T. Q. Hoang, V. Pikov, E. Meng, *J. Neural. Eng.* **2013**, *10*, 045002.

[22] D. C. Rodger, A. J. Fong, L. Wen, H. Ameri, A. K. Ahuja, C. Gutierrez, I. Lavrov, Z. Hui, P. R. Menon, E. Meng, J. W. Burdick, R. R. Roy, V. R. Edgerton, J. D. Weiland, M. S. Humayun, Y. C. Tai, *Sens. Actuators B Chem.* **2008**, *132*, 449.

[23] T. I. Kim, J. G. McCall, Y. H. Jung, X. Huang, E. R. Siuda, Y. Li, J. Song, Y. M. Song, H. A. Pao, R. H. Kim, C. Lu, S. D. Lee, I. S. Song, G. Shin, R. Al-Hasani, S. Kim, M. P. Tan, Y. Huang, F. G. Omenetto, J. A. Rogers, M. R. Bruchas, *Science* **2013**, *340*, 211.

[24] C. Xie, J. Liu, T. M. Fu, X. Dai, W. Zhou, C. M. Lieber, *Nat. Mater.* **2015**, *14*, 1286.

[25] J. Liu, T. M. Fu, Z. Cheng, G. Hong, T. Zhou, L. Jin, M. Duvvuri, Z. Jiang, P. Kruskal, C. Xie, Z. Suo, Y. Fang, C. M. Lieber, *Nat. Nanotechnol.* **2015**, *10*, 629.

[26] L. Luan, X. Wei, Z. Zhao, J. J. Siegel, O. Potnis, C. A. Tuppen, S. Lin, S. Kazmi, R. A. Fowler, S. Holloway, A. K. Dunn, R. A. Chitwood, C. Xie, *Sci. Adv.* **2017**, *3*, e1601966.
